# Seasonal and diel acoustic presence of North Atlantic minke whales in the North Sea

**DOI:** 10.1038/s41598-019-39752-8

**Published:** 2019-03-05

**Authors:** Denise Risch, Samuel C. Wilson, Mathilde Hoogerwerf, Nienke C. F. van Geel, Ewan W. J. Edwards, Kate L. Brookes

**Affiliations:** 1 Scottish Association for Marine Science, Oban, Scotland UK; 2 Marine Scotland Science, Aberdeen, Scotland UK

## Abstract

Despite frequent records from other parts of the North Atlantic, minke whales have never been acoustically recorded in the North Sea. This study investigated the detectability of pulse trains previously associated with this species in other regions, in acoustic data from ten sites along the east coast of Scotland. Since preliminary results confirmed pulse train presence, subsequently, an automated detector was applied to these data to record the seasonal and diel presence of minke whale pulse trains. Minke whales were detected from May to November, with most detections occurring in June, July and October. No acoustic detections were made in December, January or in the month of April, whilst no data were available for February and March. This pattern of acoustic presence supports available visual data and suggested an absence of minke whales from the study area during winter. Minke whale acoustic presence showed a statistically significant diel pattern, with a detection peak during night time. This study established the acoustic detectability of minke whales in the North Sea and highlights the potential of using passive acoustic monitoring to study the seasonal presence and spatial distribution of minke whales in the North Sea and wider Northeast Atlantic.

## Introduction

The effective management and conservation of marine species requires accurate knowledge of their year-round distribution patterns and abundance. This is especially important in a world where anthropogenic activities are increasingly encroaching on species habitats, and large-scale changes in habitat suitability due to climate change are leading to species shifting their distribution patterns to adapt to changing circumstances^1,2^. Large-scale surveys for marine mammal abundance and distribution often use primarily visual methods, and are conducted in daylight and during summer months when weather conditions are favourable, leaving large data gaps about habitat use at different times of day, seasonal movement and winter distribution for many species^3^. However, recent studies have shown the benefit of using long-term passive acoustic monitoring (PAM) to address this lack of data and document year-round distribution patterns and large-scale movements of baleen whales^4–6^.

The minke whale (*Balaenoptera acutorostrata*) is the smallest baleen whale species in the Northeast Atlantic, ranging from the Barents Sea to the west African continental shelf^6,7^. In UK coastal waters and the North Sea, minke whales have been visually observed mainly from April to October^8–10^, although sightings have been documented year-round^11,12^. As there is less survey effort in winter, it is unclear to what extent this occurrence pattern reflects observation effort. Off the coast of Scotland, minke whale visual sightings peak from July to August and have been shown to be related to meso-scale oceanographic features which likely link to increased foraging opportunities^13,14^. In the southern North Sea, minke whales are less common, while a seasonal aggregation has been described close to the Dogger Bank, in the central North Sea^12,15^. There is evidence that minke whales undertake large-scale seasonal migrations between feeding and breeding grounds^4,16^, and there may be two separate breeding populations in the North Atlantic^17^. However, due to a lack of winter survey effort, no discrete breeding grounds have been identified yet, and there is limited understanding of the winter distribution and occurrence of this species for the entire North Atlantic.

North Atlantic minke whales are currently listed as a species of Least Concern under the IUCN Red List^7^. Nonetheless, the species is still commercially hunted in areas of its summer range^18^. In addition, like most species of marine mammals, it is subject to indirect takes in fisheries^19,20^. Data from the UK stranding scheme suggest that entanglement in fishing gear might be a significant cause of death in baleen whales, including minke whales, in Scotland, with over 50% of examined cases between 1990 and 2010 having been documented as entanglement cases^21^. In 2015, three out of four stranded minke whales investigated at post mortem in Scotland died as a consequence of entanglement^22^. North Atlantic minke whales are also exposed to a variety of other anthropogenic threats, including ship-strike, which was determined as a cause of death in 10% of all recorded UK minke whale strandings from 1990–2010^23^. Chemical and noise pollution, and associated degradation and loss of habitat are additional threats to minke whales throughout their range^24^.

Like all cetaceans in European waters, minke whales are protected through the EU Habitats Directive, and associated national legislation. Population estimates and assessments in relation to *favourable conservation status* are primarily based on decadal, large-scale visual surveys, such as SCANS I, II and III^3^. The latest abundance estimate for the North Sea is 8,900 animals (CV = 0.24), based on shipboard and aerial surveys undertaken in the summer of 2016^3^. Information on minke whale abundance and distributions on finer spatial and time scales is relatively uncommon or very site specific.

In addition to light and weather constrains, visual detection of minke whales at sea can be difficult due to small group sizes and the species’ often shy and cryptic behaviour. Thus, alternative methods, such as PAM, may significantly improve studies of the ecology of this species, inform environmental impact assessments (EIAs) and help design and effectively monitor marine protected areas (MPAs).

The sounds that minke whales produce are known to vary across their geographic range. In the past, series of clicks in the 5–6 kHz range, as well as lower frequency downsweeps (118–80 Hz) have been attributed to the species in the North Atlantic^25,26^. These call types have typically been described during short-term studies, in specific locations and not regularly been documented across habitats. Longer-term time series of these calls are to our knowledge currently not available. In contrast, low-frequency pulse trains (50–400 Hz) with varying inter-pulse interval structure were recorded in the presence of minke whales in the Caribbean, and subsequently also documented and further described from recordings made in Massachusetts Bay^27,28^. Based on these latter data, an automated pulse train detector was developed to investigate occurrence and large-scale movement and migration patterns of the species in the western North Atlantic^4,28,29^. However, when interpreting patterns of call occurrence it is important to keep in mind, that the behavioural significance of these vocalisations and whether they are specific to sex, age, recording site or season is currently unknown.

The main goal of the current study was to confirm the presence of minke whale pulse trains in a known summer feeding ground in the northern North Sea, and secondly to test the performance of the automated detector developed for minke whale pulse trains from the western North Atlantic, on long-term recordings from the North Sea. Finally, detection results were investigated to determine seasonal and diel occurrence patterns of minke whales and explore the possibility to improve, and spatio-temporally extend, current monitoring of this species in UK and adjacent Northeast Atlantic areas using PAM.

## Results

### Detector performance

The manually reviewed truth dataset consisted of a total of 2,400 hours, containing 322 candidate detection hours. For this subset of data from across the whole array, the detector recall and precision values were 74% and 20%, respectively. The false positive rate was 11%. Although not evaluated in detail here, the high number of false detections leading to the low precision values, mainly consisted of vessel noise and noise from seismic surveys.

### Minke whale pulse train detection and spatial distribution

**Figure 1:**
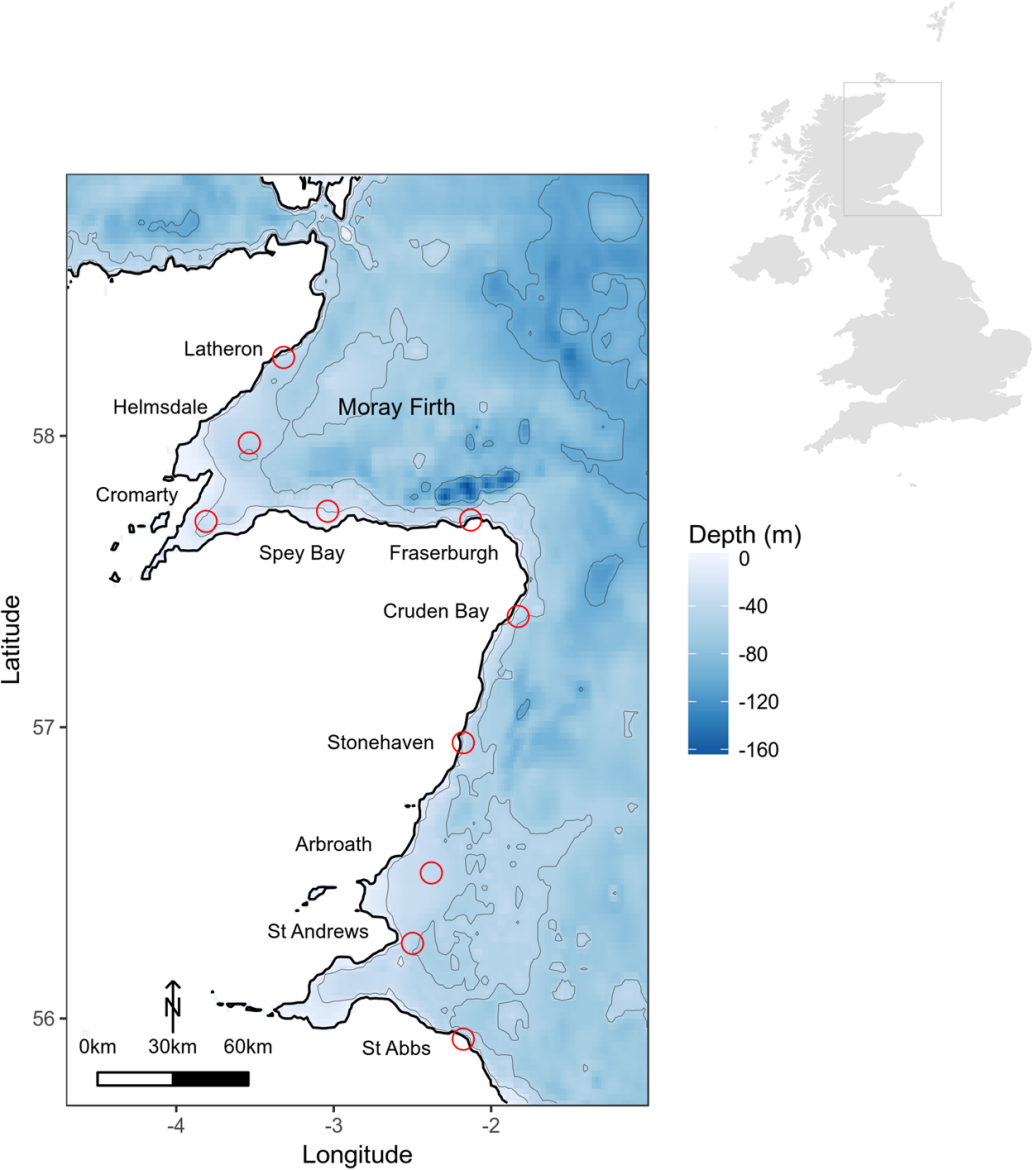
Map of the study area in the Moray Firth and along the east coast of Scotland, with red circles marking recording locations. Map in upper right corner illustrates the location of the study area along the Scottish North Sea coast.

**Figure 2:**
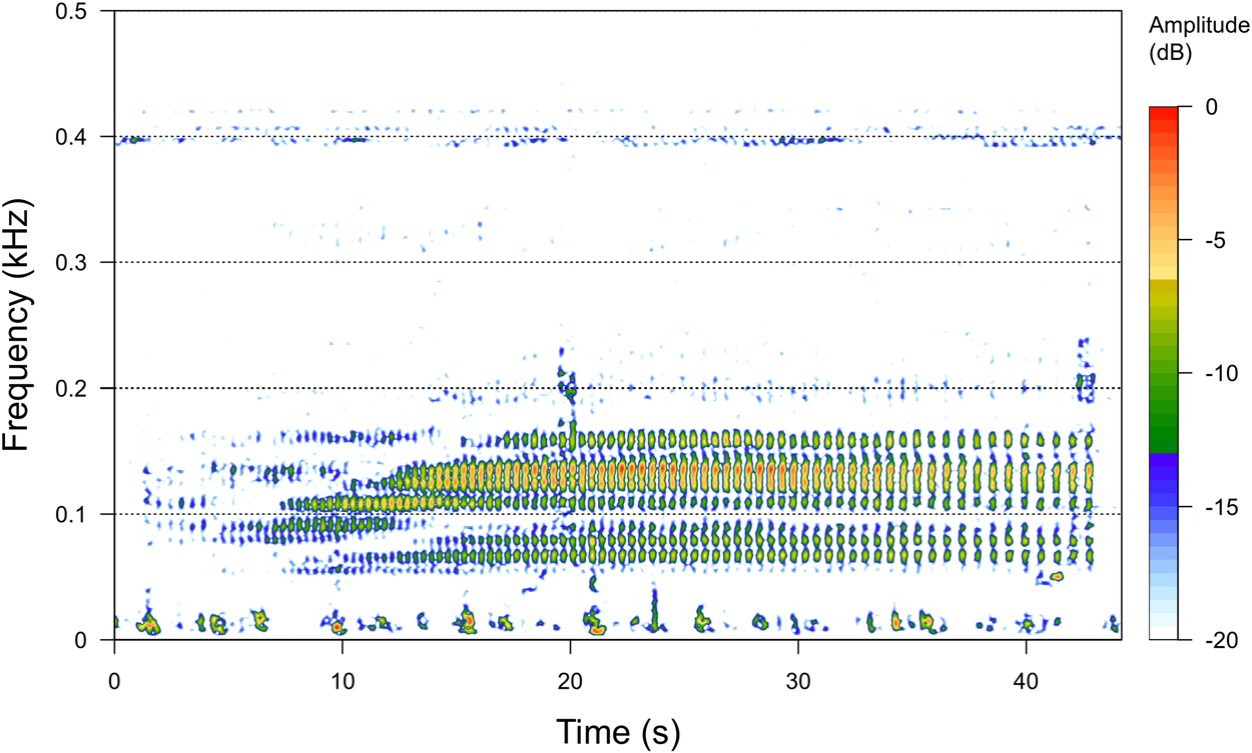
Spectrogram of a slow-down pulse train recorded off Helmsdale. Spectrogram parameters: fast Fourier transform (FFT) size: 1024 points, overlap: 95%, sample rate: 2000, resolution: 2 Hz and 128 ms. Amplitude scale is relative.

A total of 32,830 hours of acoustic recordings were collected from May to November 2016 across all ten recording sites in the Moray Firth and along the East Coast of Scotland (Fig. 1), yielding 340 manually verified positive detection hours. Pulse train characteristics were similar to those described in the Northwest Atlantic^27,28^, with peak frequencies between 50–150 Hz and pulse train durations ranging from 20–60 seconds (Fig. 2). Most pulse trains observed in this data set were slow-down pulse trains, showing a decrease in inter-pulse interval over time^28^.

**Figure 3:**
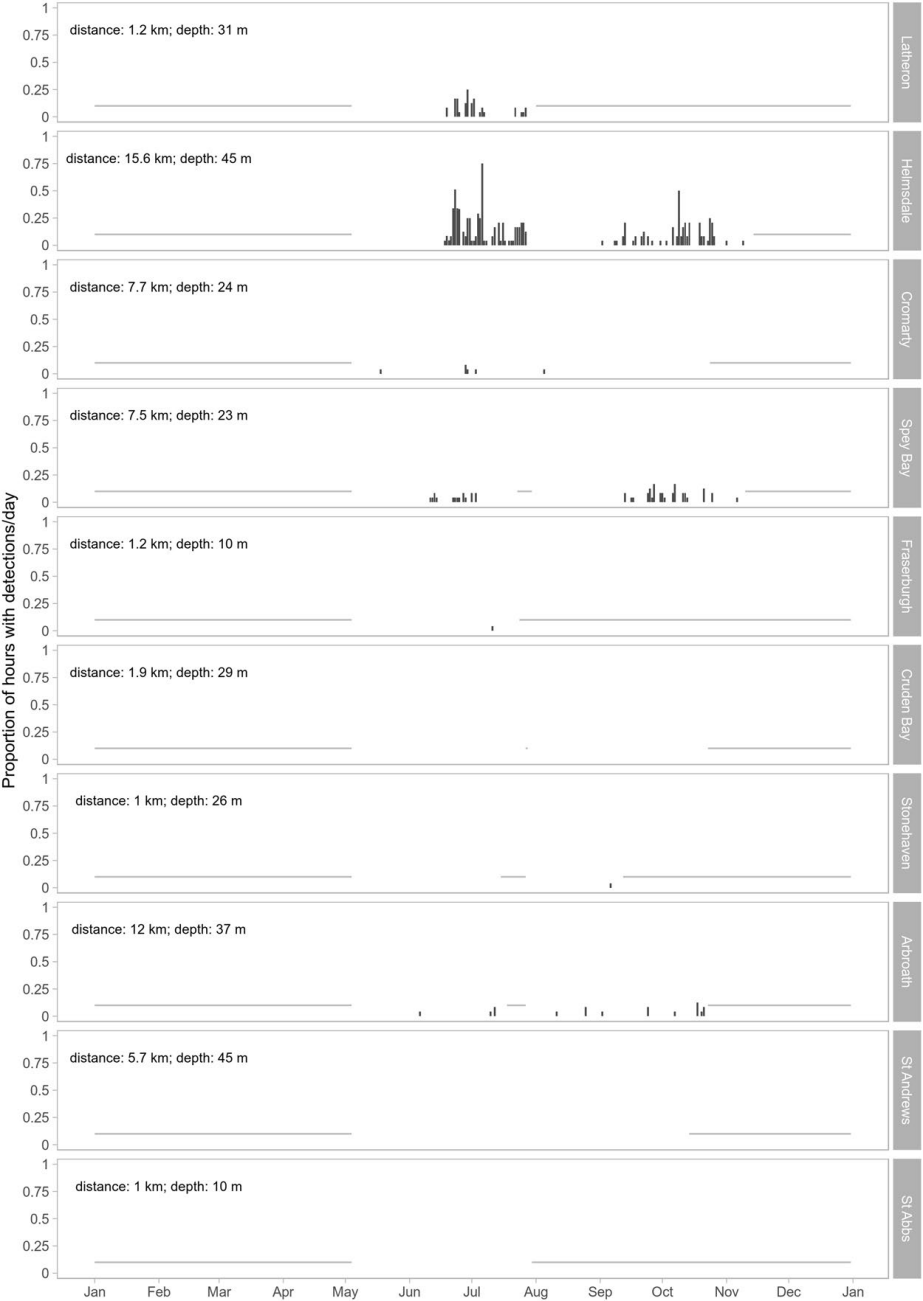
Seasonal distribution of minke whale pulse trains in 2016, expressed as the proportion of hours per day with detections for each recording location (ordered from north to south; see Fig. 1). Missing data indicated by grey lines, and distance to shore and depth given for each recording location.

Minke whale pulse trains were detected at seven of the ten recording sites. Most detection hours occurred in the central and northern Moray Firth, in particular at the Latheron, Helmsdale and Spey Bay sites (Table 1, Fig. 3). With the exception of the Arbroath site (17 detection hours), fewer or no detections were recorded along the Scottish east coast outwith the Moray Firth (Table 1, Fig. 3). In addition, most detections were made at locations with water depth equal to or greater than 23 m (Table 1).

**Table 1:** Summary of recording locations, water depth, recording times, detection hours and hours analysed in 2016 across the whole passive acoustic array.

Location	Lat	Lon	Depth (m)	Start	End	Hours analysed	Detection Hours
Latheron	58.27	−3.32	31	07/05/2016	31/07/2016	1,986	37
Helmsdale	57.97	−3.54	45	07/05/2016	11/13/2016	4,434	226
Cromarty	57.71	−3.81	24	07/05/2016	23/10/2016	4,054	6
Spey Bay	57.74	−3.04	23	07/05/2016 31/07/2016	22/07/2016 09/11/2016	4,275	52
Fraserburgh	57.71	−2.13	10	07/05/2016	23/07/2016	1,858	1
Cruden Bay	57.38	−1.83	29	07/05/2016 28/07/2016	26/07/2016 22/10/2016	3,999	0
Stonehaven	56.95	−2.18	26	07/05/2016 28/07/2016	14/07/2016 11/09/2016	2,740	1
Arbroath	56.49	−2.38	37	07/05/2016 28/07/2016	17/07/2016 22/10/2016	3,783	17
St Andrews	56.26	−2.49	45	07/05/2016	13/10/2016	3,818	0
St Abbs	55.93	−2.18	10	07/05/2016	29/07/2016	1,883	0
**Total**						**32,830**	**340**

### Seasonal and multi-year presence

**Figure 4:**
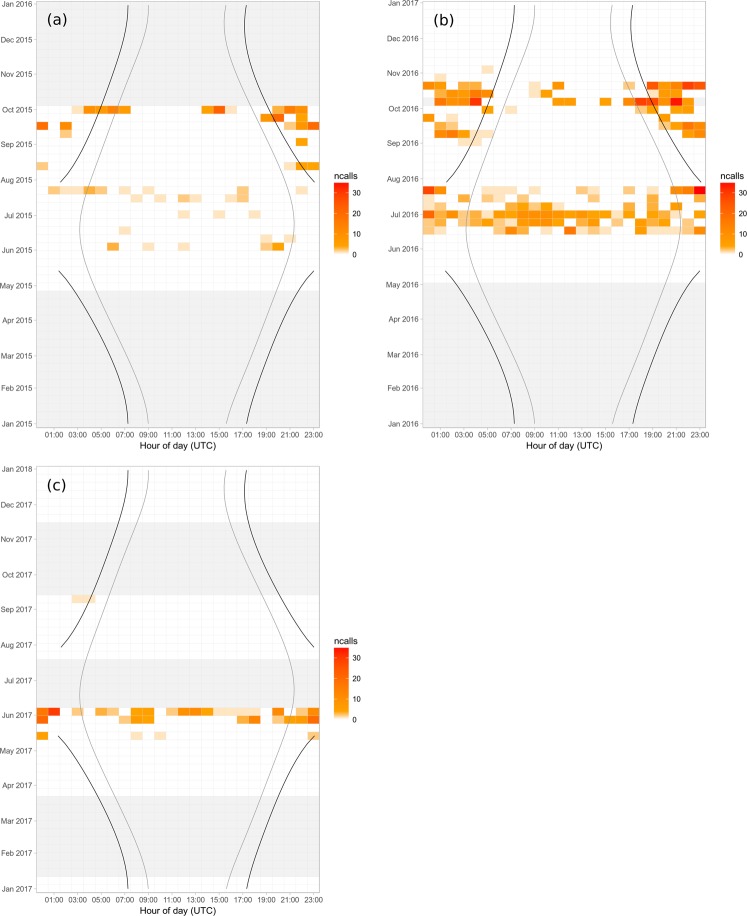
Number of total identified detections of minke whales (ncalls) at the Helmsdale recording site, aggregated by week (y-axis, tick marks indicate start of month) and hour of day (x-axis, tick marks indicate mid-hour), for (**a**) 2015, (**b**) 2016, (**c**) 2017. Black and grey lines indicate dawn/dusk and sunrise/sunset times, respectively. This high latitude recording area experiences all-night nautical twilight from May to August as indicated by the broken black dawn/dusk lines. The ‘sun-methods’ function of the R^46^ *maptools* library^47^ was used to determine times for sunrise, sunset, dawn and dusk based on nautical twilight (defined as sun altitude between 0 and 12°) for each day. Light grey shaded areas indicate periods of missing data. Data for January 2018 were omitted from this graph for ease of presentation and since no detections were made during this month.

Minke whale pulse trains were detected in all months for which data were available across all sites (May to November 2016) (Fig. 3). Most detections were made in June and July (95 and 109 detection hours, respectively), with a second peak in occurrence during the month of October (86 detection hours). A total of 328 detection hours were recorded in multi-year data (May 2015–January 2018) from Helmsdale, the site with most detections in 2016 (Fig. 4, Table 2). The general pattern of seasonal presence from the end of May to early November was repeated in the three consecutive years. However, in part due to missing data, the bimodal distribution in occurrence observed in 2016, was not observed in 2015 or 2017/18 (Fig. 4). Whilst no data were available for February and March in any of the recording years, no detections were made during available winter months (December and January) (Fig. 4).

**Table 2:** Summary of detection hours and hours analysed from May 2015 to January 2018 for the Helmsdale recording site.

Year	Start	End	Hours analysed	Detection Hours
2015	01/05/2015	05/10/2015	3,735	60
2016	07/05/2016	31/12/2016	5,431	226
2017/18	01/01/201730/03/2017 28/07/2017 21/11/2017	09/01/2017 11/06/2017 16/09/2017 28/01/2018	4,824	42
**Total**			**13,990**	**328**

### Diel pattern

**Figure 5:**
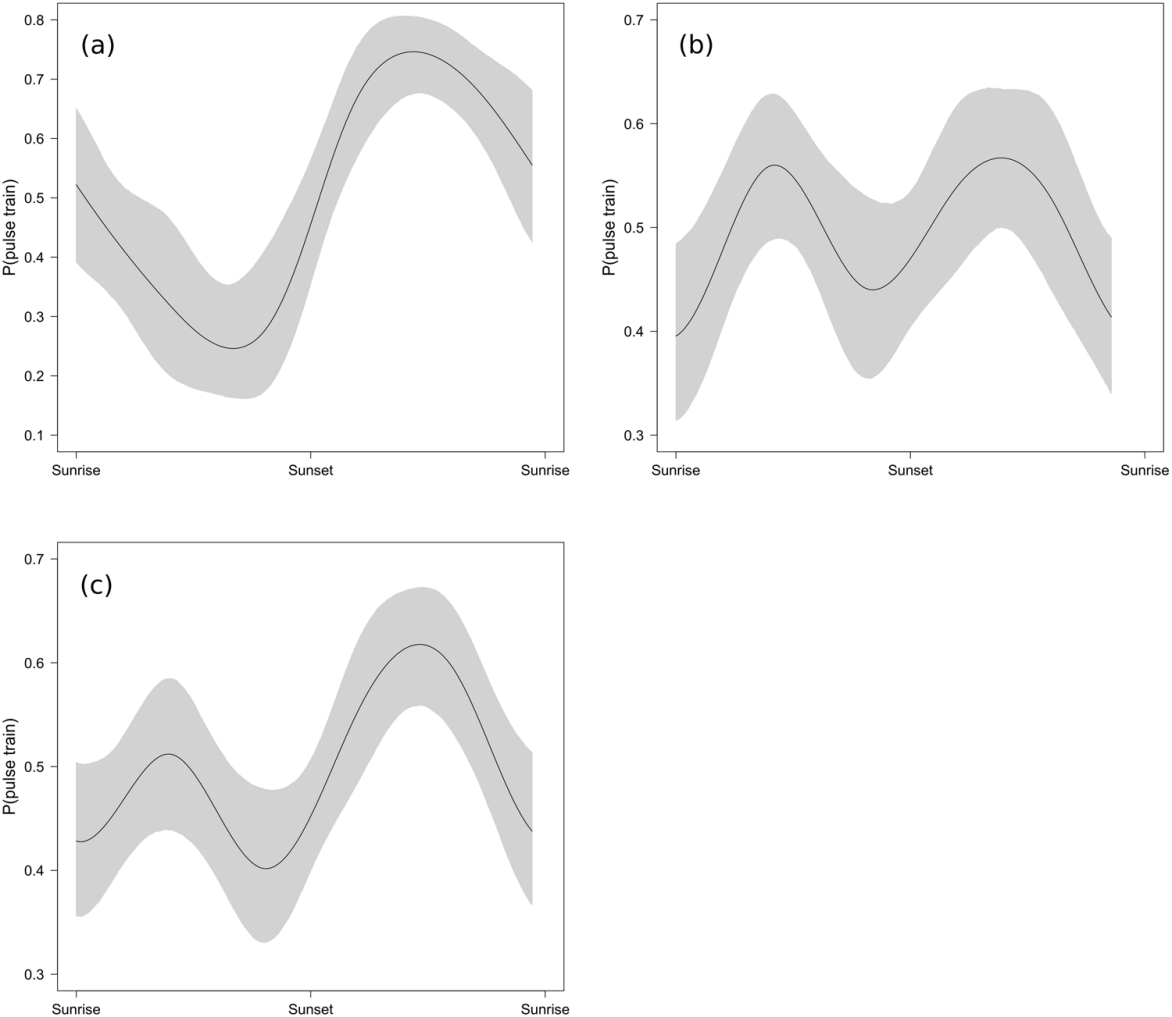
Results of binary GAM with GEE models for the presence/absence of minke whale pulse trains in each hour of recording, estimating the relationship between the response variable and a diel cycle index value, based on sunrise/sunset data and calculated for each hour (see Methods). Models were run for all multi-year data (2015–2018) collected at the Helmsdale recording site (**c**), and separately for autumn-spring (**a**) and summer (**b**) data. Summer was defined as the period between May 20^th^ and July 22^nd^, when the study area experiences all-night nautical twilight. The light grey shaded areas represent 95% confidence intervals.

Minke whale pulse train occurrence showed a clear diel pattern especially during autumn (September to November, Fig. 4), with a majority of detections recorded during night time and nautical twilight. The GAM-GEE models run on the full data set corroborated this finding, revealing a significant relationship between minke whale presence and the diel cycle index (Wald test: df = 4, Χ^2^ = 17.1, p = 0.0018), with a peak in detections between sunset and sunrise (Fig. 5). When running models separately for the summer period with all-night twilight (May 20^th^–July 22^nd^) and the rest of the year, both models showed a significant relationship of the diel cycle index with minke whale presence. However, the relationship during summer was considerably weaker (Wald test: df = 4, Χ^2^ = 9.7, p = 0.046), than during the rest of the year when the night-time peak of detection is more pronounced (Wald test: df = 4, Χ^2^ = 42.6, p < 0.0001, Fig. 5).

## Discussion

The results of this study are to our knowledge the first confirmation of minke whale pulse train detection in the well studied Moray Firth summer feeding ground and adjacent North Sea waters^9,13,14^. Minke whale pulse trains were detected at several sites of this shallow, coastal PAM array. While minke whale pulse trains have been described on one occasion from the west coast of Scotland^30^, few other acoustic recordings of this species from UK coastal waters exist. However, these vocalisations have recently been described in detail from the Northwest Atlantic^28^ and have also been detected further north in the Northeast Atlantic^31^, and on several large-scale acoustic arrays in the central North Atlantic^32,33^. Low-frequency minke whale pulse trains recorded in the current study closely resemble those documented in the central and Northwest Atlantic^28,33^. A more detailed study is necessary to elucidate the potential for geographic variation in call types or call repertoire. However, notwithstanding the potential for such regional variability, the similarities between acoustic signals recorded in this study with calls recorded in other parts of the Atlantic, allowed the unequivocal identification of species-specific minke whale vocalisations and assessment of spatio-temporal distribution patterns based on these acoustic data.

The minke whale pulse train detector used in this study was originally developed for a data set from the western North Atlantic^29^. Given the paucity of minke whale vocalisation records from the eastern North Atlantic, it was unclear how the detector would perform on data from this region. *Post-hoc* data analysis revealed that the data set was characterised by many hours of seismic survey activity and shipping noise at some recording locations. Comparatively high levels of shipping noise in this region have also been documented in previous years^34^. These background noise conditions likely influenced detector performance and resulted in the low precision value (20%) observed. However, the false alarm rate of the detector was considerably higher than that observed in the Northwest Atlantic, despite those data also showing high levels of shipping noise^35,36^. Thus, the higher false alarm rate in the current study was most likely related to the presence of seismic pulse trains, which were often misclassified as minke whale pulse trains. In the future, the number of missed and false positive detections may be reduced and general performance improved, by using a dataset from Scottish waters, including call examples and realistic ambient noise conditions from this area, to train the detector. Nevertheless, despite average performance results, the existing detector in conjunction with false detection removal during post-processing, could be used to infer realistic broader scale spatio-temporal occurrence patterns. Due to missed calls the presented data could be an underestimate of actual presence, but this bias is somewhat accounted for by binning the data by hour.

Data analysed in this study were collected from May to November 2016 across ten sites and from May 2015 to January 2018 at the Helmsdale recording site. Across the whole array and all years for the Helmsdale site, minke whale pulse trains were first detected in late May and detections generally declined at the end of October (Figs 3 and 4). This occurrence pattern supports records from visual sighting surveys, which also recorded minke whale presence from June to October in the Moray Firth^37^. It is thought that minke whales in the Moray Firth are primarily foraging on sandeel (*Ammodytes spp*.) during this period^13,38^. In 2016, detections declined during the month of August. During July and September minke whales were detected in 109 and 42 hours, respectively across the whole array, while only four hours with pulse train detections were recorded in August (Figs 3 and 4b). Additional data from Helmsdale for the years 2015 and 2017/18, also showed a reduction in detections in August and September. The cause of this seasonal reduction in acoustic detections could have been an absence of animals from the study area, suggesting seasonal movement in and out of the Moray Firth during mid- and late summer. Alternative explanations for the observed detection patterns include a switch in vocal behaviour or changes in local ambient noise or propagation conditions at this time of year.

During autumn and spring minke whale pulse train detections showed strong diel periodicity, with calling rates being lowest during light and highest during dark periods (Figs 4 and 5). Similarly strong diel patterns have been observed in Massachusetts Bay^28^. Without more knowledge on individual calling rates, the reason for the observed pattern cannot be conclusively resolved. It may be the result of higher individual calling rates, an increase of the overall number of vocalising individuals at night or a change of animal abundance from day to night time. Diel variation in baleen whale vocalisations has also been attributed to prey distribution, with reduced vocalisation rates during active feeding and an increase in vocalisations in a social context at hours of lowest prey availability^39,40^. Visual observations in the Moray Firth during day time frequently show feeding behaviour^13,14^, which might suggest that minke whales vocalise more at night, when feeding efficiency may be lower. As mentioned earlier, minke whales around Scotland, including the Moray Firth, have been shown to primarily feed on sandeel which constitutes 70% their diet^38^. Sandeel species show a strong diurnal pattern and are generally less available in the water column during night time^41^. The observed lesser diel pattern (Figs 4 and 5) during summer might then relate to differences in diel light patterns and its effect on sandeel and other prey availability, and hence minke whale feeding (and vocal) behaviour.

It is currently unclear what role minke whale pulse trains may play in the species’ ecology. However, there is circumstantial evidence to suggest that they might serve in male advertisement^28^. If minke whale pulse trains are produced in a reproductive context, another potential explanation for the observed diel distribution could be that visual displays of fitness are replaced by vocal signalling during hours of darkness^42,43^. Further investigations of minke whale vocalisations concurrent with visual observations, might help elucidate additional behavioural functions of these calls and help explain observed temporal patterns of occurrence.

Spatially, most detections were made and were of longer duration in the northern Moray Firth, while fewer and temporally more spaced detections were recorded along the east coast of Scotland (Fig. 3). These data indicate that while minke whales appear to occur along the Scottish east coast, they show a more continuous presence in the Moray Firth during summer. In 2016, the site at Helmsdale in the northern part of the Moray Firth recorded the greatest number of detections; this site was also one of the deepest recording locations and the one furthest offshore (Fig. 1, Table 1). The presence of minke whales in the deeper waters of the central and northern Moray Firth, was also demonstrated by fairly high numbers of detection hours at Latheron (Fig. 3). In contrast, the relatively low number of detection hours at Fraserburgh in the southern Moray Firth, where minke whales are frequently observed visually^13,14^ was surprising. However, these results may be partly due to the acoustic recorder being placed in very shallow waters at this site. Low frequencies (peak frequencies of minke whale pulse trains are between 150–200 Hz^28^; Fig. 2) propagate poorly in shallow waters, as these effectively act as high-pass filters^44^. The true presence at this location, and potentially also at other sites, might not be accurately reflected using the available data recorded with an array originally designed to study coastal bottlenose dolphin distribution. This result highlights the importance of taking recorder location and local environmental conditions into account when interpreting results from passive acoustic recordings.

When using PAM for future monitoring of minke whales (e.g., in the Southern Trench MPA proposal area in the southern Moray Firth), care should be taken to place the PAM system in waters deep enough to ensure effective detection of the low frequency vocalisations produced by minke whales. Previous surveys of minke whales are largely based upon visual observations, be they from land, ship or from aircraft. These surveys suffer from low encounter rates and the case for future MPA proposals could be greatly strengthened though the more widespread use of PAM for monitoring minke whales. The long periods of observation by multiple acoustic recorders could greatly improve the confidence in minke whale distribution, and provide a cost-effective method for long-term monitoring of MPAs for minke whales. Similarly, PAM offers a contribution to the recurrent need to undertake risk assessments of offshore developments in relation to impacts on marine mammal populations. PAM should enable clarification of the relative importance of different potential development sites to minke whale populations, and give guidance to possible spatially-based impact mitigation strategies. The data from the current project were largely confined to recording sites within 15 km of the shore. However, our results do suggest that deeper waters in the central Moray Firth, might be more important for minke whales than previously thought based on visual data alone.

At a broad scale, the detection of minke whale pulse trains in the Moray Firth and North Sea supports visual distribution patterns for this region and confirms the feasibility of using PAM to monitor this species in Scottish, wider UK and Northeast Atlantic waters. Future work should include the improvement of the performance of the automated detector, using regional pulse train examples and distinguishing these against local ambient noise data. More work is also needed on the description of the full vocal repertoire for the species in UK coastal waters, as well as the behavioural context of minke whale vocalisations in general, including caller identity, source level and calling rates. With such additional knowledge, PAM may then significantly improve current conservation, monitoring and management efforts for minke whales in UK waters.

## Methods

### Acoustic data collection

The East Coast Marine Mammal Acoustic Study (ECOMMAS) has been monitoring acoustic presence of cetaceans and ambient noise in the Moray Firth and along the east coast of Scotland (http://marine.gov.scot/information/east-coast-marine-mammal-acoustic-study-ecommas). As part of this effort, acoustic broadband data have been collected at ten different recording sites since 2013. The original aim for this long-term study was to better understand coastal bottlenose dolphin movement, which informed the placement of recorders at varying distances (up to ~15 km) from the low tide line and at depths ranging from approximately 10–45 m (Fig. 1, Table 1). Acoustic recorders (SM2M, Wildlife Acoustics) were typically moored 3–5 m above the sea floor and programmed to record at a sample rate of 96 kHz, with 12 dB gain and a 10/20 minutes on/off duty cycle. Each recorder was equipped with a HTI-94 hydrophone (sensitivity: -168 dB re 1 V/μPa, flat (+/-1 dB) frequency response from 2 Hz to 40 kHz).

Minke whale acoustic presence and spatial distribution was investigated by analysing available recordings from all 10 recording sites for the period from May to November 2016 (total across all sites: 32,827 hours, Table 1). Since most detections in 2016 were collected at the Helmsdale recording site (Fig. 1, Table 1), multi-year data (2015–2018), including additional data for December 2016, were analysed for this site to extend recordings into winter months and investigate inter-annual variability, as well as diel patterns (total: 13,990 hours, Table 2).

### Analysis

#### Data preparation and initial acoustic analysis

To focus the analysis on low frequencies for detection of minke whale pulse train presence, all data were initially downsampled to a sample rate of 2 kHz using the Decimator module in PAMGuard^45^ and applying a low-pass 4^th^ order Butterworth filter. A random subsample of 100 days, 10 days from each site, collected in 2016, was then reviewed visually and aurally, using spectrograms (fast Fourier transformation [FFT] size: 2048 points, 75% overlap, Hanning window) created in XBAT^46^.

#### Automated detection process

After confirmation of minke whale pulse train presence at several recording sites, an automated detection algorithm, originally developed for pulse trains from the western North Atlantic, was used to analyse data from the whole ECOMMAS array. The detector was run in a batch process on sound files loaded in XBAT. The automatic detection consisted of a multi-stage process based on spectrogram intensity binarisation, energy projection, feature extraction and finally pulse train detection and classification^29^. The detector design and performance for data from the western Atlantic is described in more detail in Popescu *et al*.^29^.

#### Ground truth and detector evaluation

The 100 day data set from 2016, yielded 2,400 analysis hours (analysis hour referring to the 20 minutes of recording in each hour) that were manually reviewed by experienced data analysts (DR, MH, SW). Analysts recorded the presence of all true positive (TP), false negative (missed) (FN), and false positive (FP) detection hours. The results of this analysis were used to assess overall detector performance under varying noise conditions. The *recall* or true positive rate (TPR) of the detector is the percentage of manually identified detection hours which were also detected automatically, i.e. the number of true detection hours by the detector divided by the total number of true detection hours as identified by data analysts (TP/(TP + FN)). The *precision* of the detector is the percentage of correct automatic detections, i.e. the number of true detection hours divided by the total number of detection hours by the detector (TP/(TP + FP)). Finally, the false positive rate (FPR) was calculated as the number of false positive detection hours, as classified by the automated detector, divided by the total number of true negative detection hours identified by data analysts (FP/(TP + FP). While a high recall rate indicates a high detection efficiency and few missed calls, a high precision value and low false positive rate indicate a low false alarm rate.

#### Spatial and seasonal distribution patterns in 2016

After manual review of all detection hours recorded from May to November 2016, all false positive detection hours were removed from the final dataset, resulting in a time-series of minke whale pulse train presence at a 1-hour resolution for each of the ten recording sites. These data were then corrected for recording effort by dividing the total number of detection hours by the total number of effort hours for each day.

#### Multi-year seasonal and diel detection patterns

Analysis of data collected from May to November 2016, found most minke whale detections at the Helmsdale recording site (Table 1, Fig. 3). To explore diel and seasonal patterns across years, an extended data series from April 2015 to January 2018 was analysed only for this location. The automated detector was run as described above, acoustic detection hours were manually reviewed and pulse train counts for each hour with true positive detections enumerated, while false positive detections were removed. The number of true pulse train detections were then grouped by hour of day (using GMT), aggregated by recording week and plotted against time of day, dawn, sunrise, sunset and dusk to investigate seasonal and diel patterns in pulse train occurrence (Fig. 4). The ‘sun-methods’ function of the R^47^ *maptools* library^48^ was used to determine times for sunrise, sunset, as well as dawn and dusk based on nautical twilight (defined as sun altitude between 0 and 12°) for each day.

Diel trends in minke whale detections from the multi-year Helmsdale data set were further investigated by modelling the hourly presence/absence of minke whale pulse trains in relation to diel cycle index values, scaled to account for seasonal variation in day length. The model was run on the whole data set, as well as separately for the period of all-night twilight during summer (May 20^th^–July 22^nd^) and the rest of the year (autumn to spring) when full darkness did occur. For each hour, the diel cycle index was calculated based on sunrise/sunset data, where index values of 0 and 1 corresponded to the hour of sunrise and 0.5 represented the hour in which sunset occurred. A binomial generalised additive model (GAM) was fitted with the diel cycle index as explanatory variable, an independent correlation structure and a logit link function, and using generalised estimating equations (GEEs) to account for temporal autocorrelation in the model residuals, following Pirotta *et al*.^49^. While GAMs assume independence among model residuals, the GEE method models the correlation within specified ‘blocks’ of data, assuming independence between blocks^49,50^. A block size of 24 hour was chosen based on examination of autocorrelation function (ACF) plots of the model residuals. The significance of the diel cycle was assessed using a Wald chi-squared test and the relationship between diel cycle and the probability of minke whale hourly presence visualised using a partial residual plot (Fig. 5). Models were built using the R *geepack* and *splines* libraries^51^, and the *nlme* and *mgcv* packages^52,53^.

## Data Availability

The datasets generated during and/or analysed during the current study can be made available upon reasonable request.

## References

[1] Lambert E (2011). Quantifying likely cetacean range shifts in response to global climatic change: implications for conservation strategies in a changing world. Endanger. Species Res..

[2] Ramp C, Delarue J, Palsbøll PJ, Sears R, Hammond PS (2015). Adapting to a warmer ocean—seasonal shift of baleen whale movements over three decades. PLoS One.

[3] Hammond, P. S. *et al*. Estimates of cetacean abundance in European Atlantic waters in summer 2016 from the SCANS-III aerial and shipboard surveys. 39; https://synergy.st-andrews.ac.uk/scans3/files/2017/04/SCANS-III-%0Adesign-based-estimates-2017-04-28-final.pdf (2017).

[4] Risch D (2014). Seasonal migrations of North Atlantic minke whales: novel insights from large-scale passive acoustic monitoring networks. Mov. Ecol..

[5] Balcazar NE (2017). Using calls as an indicator for Antarctic blue whale occurrence and distribution across the southwest Pacific and southeast Indian Oceans. Mar. Mammal Sci..

[6] van Waerebeek K (1999). Spatial and temporal distribution of the minke whale *Balaenoptera acutorostrata* (Lacepede, 1804), in the southern northeast Atlantic Ocean and the Mediterranean Sea, with reference to stock identity. J. Cetacean Res. Manag..

[7] Reilly, S. B. *et al*. Balaenoptera acutorostrata. *The IUCN Red List of Threatened Species 2008: e.T2474A9444043*. http://www.iucnredlist.org (2008).

[8] Northridge SP, Tasker ML, Webb A, Williams JM (1995). Distribution and relative abundance of harbour porpoises (*Phocoena phocoena* L.), white-beaked dolphins (*Lagenorhynchus albirostris* Gray), and minke whales (*Balaenoptera acutorostrata* Lacepède) around the British Isles. ICES J. Mar. Sci. J. du Cons..

[9] Weir CR, Stockin KA, Pierce GJ (2007). Spatial and temporal trends in the distribution of harbour porpoises, white-beaked dolphins and minke whales off Aberdeenshire (UK), north-western North Sea. J. Mar. Biol. Assoc. United Kingdom.

[10] Dolman SJ, Hodgins NK, Macleod CD, Pierce GJ, Weir CR (2013). Harbour porpoises (*Phocoena phocoena*) and minke whales (*Balaenoptera acutorostrata*) observed during land-based surveys in The Minch, north-west Scotland. J. Mar. Biol. Assoc. UK.

[11] Macleod K (2004). Seasonal distribution of minke whales *Balaenoptera acutorostrata* in relation to physiography and prey off the Isle of Mull, Scotland. Mar. Ecol. Prog. Ser..

[12] Reid, J. B., Evans, P. G. H. & Northridge, S. P. *Atlas of cetacean distribution in north-west European waters*. (Joint Nature Conservation Committee (JNCC), 2003).

[13] Tetley MJ, Mitchelson-Jacob EG, Robinson KP (2008). The summer distribution of coastal minke whales (*Balaenoptera acutorostrata*) in the southern outer Moray Firth, NE Scotland, in relation to co-occurring mesoscale oceanographic features. Remote Sens. Environ..

[14] Robinson KP, Tetley MJ, Mitchelson-Jacob EG (2009). The distribution and habitat preference of coastally occurring minke whales (*Balaenoptera acutorostrata*) in the outer southern Moray Firth, northeast Scotland. J. Coast. Conserv.

[15] de Boer M (2010). Spring distribution and density of minke whale *Balaenoptera acutorostrata* along an offshore bank in the central North Sea. Mar. Ecol. Prog. Ser..

[16] Skaug HJ, Øien N, Schweder T, Bøthun G (2004). Abundance of minke whales (*Balaenoptera acutorostrata*) in the Northeast Atlantic: variability in time and space. Can. J. Fish. Aquat. Sci..

[17] Anderwald P (2011). Possible cryptic stock structure for minke whales in the North Atlantic: Implications for conservation and management. Biol. Conserv..

[18] Robards MD, Reeves RR (2011). The global extent and character of marine mammal consumption by humans: 1970-2009. Biol. Conserv..

[19] Read AJ (2008). The looming crisis: interactions between marine mammals and fisheries. J. Mammal..

[20] Reeves RR, McClellan K, Werner TB (2013). Marine mammal bycatch in gillnet and other entangling net fisheries, 1990 to 2011. Endanger. Species Res.

[21] Northridge, S., Cargill, A. & Coram, A. Entanglement of minke whales in Scottish waters; an investigation into occurrence, causes and mitigation. 1–57 (2010).

[22] Deaville, R. *et al*. Annual report for the period 1st January 2005-31st December 2015. UK Cetacean Strandings Investigation Programme (CSIP). http://randd.defra.gov.uk/Document.aspx?Document=14001_FINALUKCSIPAnnualReport2015.pdf (2016).

[23] Deaville, R. & Jepson, P. Final report for the period 1st January 2005-31st December 2010. UK Cetacean Strandings Investigation Programme (CSIP). http://randd.defra.gov.uk/Document.aspx?Document=11149_FINALUKCSIPAnnualReport2011(2).pdf (2011).

[24] Clapham PJ, Young SB, Brownell RL (1999). Baleen whales: conservation issues and the status of the most endangered populations. Mamm. Rev.

[25] Beamish P, Mitchell E (1973). Short pulse length audio frequency sounds recorded in the presence of a Minke whale (*Balaenoptera acutorostrata*). Deep Sea Res. Oceanogr. Abstr.

[26] Edds-Walton P (2000). Vocalizations of minke whales *Balaenoptera acutorostrata* in the St. Lawrence Estuary. Bioacoustics.

[27] Mellinger DK, Carson CD, Clark CW (2000). Characteristics of minke whale (*Balaenoptera acutorostrata*) pulse trains recorded near Puerto Rico. Mar. Mammal Sci..

[28] Risch D (2013). Minke whale acoustic behavior and multi-year seasonal and diel vocalization patterns in Massachusetts Bay, USA. Mar. Ecol. Prog. Ser..

[29] Popescu, M. *et al*. Bioacoustical periodic pulse train signal detection and classification using spectrogram intensity binarization and energy projection. *ICML 2013 Workshop on Machine Learning for Bioacoustics* (2013).

[30] Swift, R., Menhenett, C. & Gordon, J. C. D. Feasibility study using sonobuoys to study cetecean acoustic behaviour in the coastal waters in the Inner Hebrides, Scotland. *10th Annual Conference of the European Cetacean Society* (1996).

[31] Folkow LP, Blix AS (1991). Norwegian whale sighting and acoustic surveys in the Atlantic Ocean during the winter of 1989/90. Rep. Int. Whal. Comm.

[32] Clark CW, Gagnon GJ (2004). Low-frequency Vocal Behaviors of Baleen Whales in the North Atlantic: Insights from Integrated Undersea Surveillance System Detections, Locations, and Tracking from 1992 to 1996. US Navy J. Underw. Acoust..

[33] Nieukirk SL, Stafford KM, Mellinger DK, Dziak RP, Fox CG (2004). Low-frequency whale and seismic airgun sounds recorded in the mid-Atlantic Ocean. J. Acoust. Soc. Am..

[34] Merchant ND (2016). Underwater noise levels in UK waters. Sci. Rep.

[35] Hatch LT, Clark CW, van Parijs SM, Frankel AS, Poniraiks DW (2012). Quantifying loss of acoustic communication space for right whales in and around a U.S. National Marine Sanctuary. Conserv. Biol..

[36] Haver SM (2018). Monitoring long-term soundscape trends in US Waters: The NOAA/NPS Ocean Noise Reference Station Network. Mar. Policy.

[37] Robinson KP (2007). The summer distribution and occurrence of cetaceans in the coastal waters of the outer southern Moray Firth in northeast Scotland (UK). Lutra.

[38] Pierce GJ, Santos MB, Reid RJ, Patterson IAP, Ross HM (2004). Diet of minke whales Balaenoptera acutorostrata in Scottish (UK) waters with notes on strandings of this species in Scotland 1992–2002. J. Mar. Biol. Assoc. United Kingdom.

[39] Baumgartner MF, Fratantoni DM (2008). Diel periodicity in both sei whale vocalization rates and the vertical migration of their copepod prey observed from ocean gliders. Limnol. Oceanogr..

[40] Širović A, Williams LN, Kerosky SM, Wiggins SM, Hildebrand JA (2013). Temporal separation of two fin whale call types across the eastern North Pacific. Mar. Biol..

[41] Freeman S, Mackinson S, Flatt R (2004). Diel patterns in the habitat utilisation of sandeels revealed using integrated acoustic surveys. J. Exp. Mar. Bio. Ecol.

[42] Au WWL, Mobley J, Burgess WC, Lammers MO, Nachtigall PE (2000). Seasonal and diurnal trends of chorusing humpback whales wintering in waters off western Maui. Mar. Mammal Sci..

[43] Munger LM, Wiggins SM, Moore SE, Hildebrand JA (2008). North Pacific right whale (*Eubalaena japonica*) seasonal and diel calling patterns from long-term acoustic recordings in the southeastern Bering Sea, 2000-2006. Mar. Mammal Sci.

[44] Richardson, W. J., Greene, C. R., Malme, C. I. & Thomson, D. H. *Marine mammals and noise*. (Academic Press, 1995).

[45] Gillespie D (2008). PAMGUARD: Semiautomated, open source software for real-time acoustic detection and localisation of cetaceans. J. Acoust. Soc. Am..

[46] Figueroa, H. K. & Robbins, M. XBAT: An Open-Source Extensible Platform for Bioacoustic Research and Monitoring. In *Computational bioacoustics for assessing biodivers*ity (eds Frommolt, K.-H., Bardeli, R. & Clausen, M.) 143–155 (Bundesamt für Naturschutz, 2008).

[47] R Core Team. R: A Language and Environment for Statistical Computing (2018).

[48] Bivand, R. & Lewin-Koh, N. maptools: Tools for reading and handling spatial objects (2013).

[49] Pirotta E, Brookes KL, Graham IM, Thompson PM (2014). Variation in harbour porpoise activity in response to seismic survey noise. Biol. Lett.

[50] Liang K-Y, Zeger SL (1986). Longitudinal data analysis using generalized linear models. Biometrika.

[51] Halekoh U, Højsgaard S, Yan J (2006). The R package geepack for generalized estimating equations. J. Stat. Softw..

[52] Pinheiro, J. *et al*. Package ‘nlme’. Linear Nonlinear Mix. Eff. Model. version 1–3 (2017).

[53] Wood, S. & Wood, M. S. Package ‘mgcv’. R Packag. Version 1 (2015).

